# *N*′-Benzoyl-*N*-*tert*-butyl-2-chloro-*N*′-{[3-(6-chloro-3-pyrid­ylmethyl)-2-nitrimino­imidazolidin-1-yl]sulfan­yl}benzo­hydrazide

**DOI:** 10.1107/S1600536809028943

**Published:** 2009-08-12

**Authors:** Jian Shang, Qing-min Wang, Run-qiu Huang, Li Chen, Jianhua Gao

**Affiliations:** a Chemistry and Biology College, Yantai University, Yantai 264005, Shandong Province, People’s Republic of China; b State Key Laboratory and Institute of Elemento-Organic Chemistry, Nankai University, Tianjin 300071, People’s Republic of China; c College of Chemistry and Life Science, Tianjin Normal University, Tianjin 300074, People’s Republic of China

## Abstract

In the title compound, C_27_H_27_Cl_2_N_7_O_4_S, the amide groups bearing the N—S group and the *tert*-butyl group have *s*–*trans* conformations. The steric size of the *tert*-butyl and [(6-chloro-3-pyrid­yl)meth­yl]imidazolidin-2-yl­idene groups cause the 2-chloro­benzoyl group and the benzyol group to be directed away from one another, forming a dihedral angle of 60.62 (17)°. The central N—N bond adopts a *gauche* conformation with a C—N—N—C torsion angle of −79.1 (2)°.

## Related literature

1-*tert*-Butyl-1,2-diacyl­hydrazines are a new class of insect growth regulators, which have been found to mimic the action of 20-hydroxyecdysone in activating the ecdysone receptor, which leads to lethal premature moulting, see: Wing (1988 ▶, 1995 ▶); Wing *et al.* (1988 ▶). 1-*tert*–Butyl-2-(4-ethyl­benzo­yl)-1-(3,5-dimethyl­benzo­yl) hydrazine (tebufenozide, RH-5992) was the first non-steroidal ecdysone agonist to be available commercially as a lepidopteran-specific insecticide, see: Dhadialla & Jansson (1999 ▶). At present, three further structural analogues are available, *viz.* methoxy­fenozide (RH-2485), halofenozide (RH-0345) and chromafenozide (ANS-118), see: Carlson *et al.* (2001 ▶); Yanagi *et al.* (2000 ▶). The *gauche* conformation of the N—N bond has been observed in other hydrazine derivatives, see: Chan *et al.* (1990 ▶); Wolfe (1972 ▶).
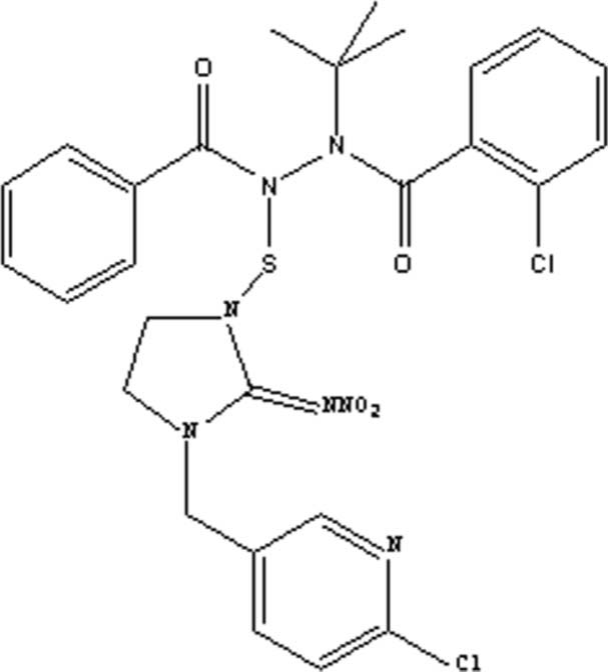


## Experimental

### 

#### Crystal data

C_27_H_27_Cl_2_N_7_O_4_S
*M* *_r_* = 616.52
Monoclinic, 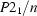

*a* = 11.2271 (19) Å
*b* = 10.1360 (17) Å
*c* = 25.660 (4) Å
β = 102.233 (2)°
*V* = 2853.7 (8) Å^3^
*Z* = 4
Mo *K*α radiation
μ = 0.35 mm^−1^
*T* = 293 K
0.32 × 0.12 × 0.10 mm

#### Data collection

Bruker SMART CCD area-detector diffractometer
Absorption correction: multi-scan (*SADABS*; Sheldrick, 1996 ▶) *T*_min_ = 0.771, *T*_max_ = 1.000 (expected range = 0.745–0.966)
15046 measured reflections
5028 independent reflections
3977 reflections with *I* > 2σ(*I*)
*R*_int_ = 0.021

#### Refinement

*R*[*F* ^2^ > 2σ(*F* ^2^)] = 0.046
*wR*(*F* ^2^) = 0.137
*S* = 1.06
5028 reflections
373 parameters
H-atom parameters constrained
Δρ_max_ = 0.78 e Å^−3^
Δρ_min_ = −0.45 e Å^−3^

### 

Data collection: *SMART* (Bruker, 1998 ▶); cell refinement: *SAINT* (Bruker, 1999 ▶); data reduction: *SAINT*; program(s) used to solve structure: *SHELXS97* (Sheldrick, 2008 ▶); program(s) used to refine structure: *SHELXL97* (Sheldrick, 2008 ▶); molecular graphics: *SHELXTL* (Sheldrick, 2008 ▶); software used to prepare material for publication: *SHELXTL*.

## Supplementary Material

Crystal structure: contains datablocks global, I. DOI: 10.1107/S1600536809028943/su2122sup1.cif

Structure factors: contains datablocks I. DOI: 10.1107/S1600536809028943/su2122Isup2.hkl

Additional supplementary materials:  crystallographic information; 3D view; checkCIF report

## Figures and Tables

**Fig. 1.**
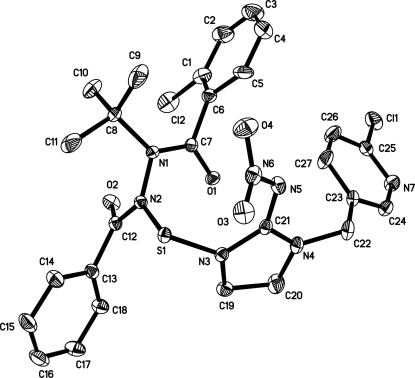
View of the molecular structure of the title compound, with displacement ellipsoids drawn at the 30% probability level (H-atoms have been omitted for clarity).

**Fig. 2.**
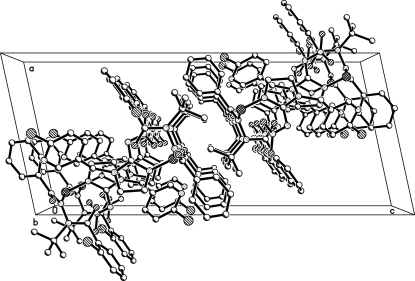
A view along the b axis, of the crystal packing of the title compound.

## supplementary crystallographic information

### Comment

1-*tert*-butyl-1,2-diacylhydrazines are a new class of insect growth regulators, which have been found to mimic the action of 20-Hydroxyecdysone in activating the ecdysone receptor, which leads to lethal premature moulting (Wing, 1988; Wing *et al.*, 1988; Wing, 1995). Among nonsteroidal ecdysone agonists, 1-*tert*-butyl-2-(4-ethylbenzoyl)-1-(3,5-dimethylbenzoyl) hydrazine (tebufenozide, RH-5992) was the first to be commercialized as a lepidopteran-specific insecticide, with a low toxicity profile towards mammals, birds and fish, as well as towards non-target arthropods such as insect pollinators, predators, and parasitoids (Dhadialla & Jansson, 1999). At present, another three new structural analogues: methoxyfenozide (RH-2485), halofenozide (RH-0345) and chromafenozide (ANS-118) have been commercialized (Carlson *et al.*,  2001; Yanagi *et al.*,  2000). Therefore, in a search for new insect growth regulators with improved biological properties and different activity spectrum, we synthesized the title compound.

The molecular structure of the title compound is shown in Fig. 1, and the crystal packing is illustrated in Fig. 2. The 2-chlorobenzoyl phenyl ring (C1-C6) is inclined to the benzyol phenyl ring (C13-C18) by 60.62 (17)°. The two carbonyl groups to which they are bonded are not coplanar with the phenyl rings. The torsion angles defined by C5—C6—C7—O1 and O2—C12—C13—C14 are -93.0 (3) and -116.9 (3)°, respectively. While the two amide functions adopt the expected planar structures, the amide bearing the N2—S1 bond has a s-*cis* conformation (N1—N2—C12—O2 = 10.9 (3)°), and that bearing the 1-*tert*-butyl group also has a s-*cis* conformation (N2—N1—C7—O1 = -1.8 (3)°). Clearly, this configurational arrangement is due to the size of the *tert*-butyl and the ((6-chloro-3-pyridyl)methyl)imidazolidin-2-ylidene substituents. The N1—N2 bond adopts a *gauche* conformation with a torsion angle of -79.1 (2)° for C7—N1—N2—C12. Such a *gauche* effect has been observed for other hydrazine derivatives (Chan *et al.*,  1990; Wolfe, 1972).

### Experimental

To a stirred solution of sulfur dichloride (0.08 mol) and dichloromethane (15 ml) was added dropwise a solution of pyridine (0.008 mol) in dichloromethane (5 ml) at 283 K. A solution of 1-*tert*-butyl-1- (2-chlorobenzoyl)-2-benzoylhydrazine (0.007 mol) in dichloromethane (5 ml) was then added at 283 K. This mixture was stirred at rt for 4 h and then added dropwise to imidacloprid sodium (0.007 mol). After the addition was complete, the reaction mixture was stirred for 6 h at rt. The solid obtained was then filtered off and the filtrate concentrated under vacuum. The residue was purified by column chromatography on silica gel using petroleum ether (60–90), dichloromethane and ethyl acetate (20:1:1 by volume) as the eluent: Yield 54%. Crystals suitable for X-ray analysis were obtained from a solution in isopropyl alcohol, by slow evaporation at room temperature.

### Refinement

All the H-atoms were placed in calculated positions and treated as riding atoms: C—H = 0.93 - 0.97 Å, with *U*_iso_(H) = 1.2*U*_eq_(C).

### Crystal data

**Table:**

C_27_H_27_Cl_2_N_7_O_4_S	*F*(000) = 1280
*M**_r_* = 616.52	*D*_x_ = 1.435 Mg m^−^^3^
Monoclinic, *P*2_1_/*n*	Mo *K*α radiation, λ = 0.71073 Å
Hall symbol: -P 2yn	Cell parameters from 4468 reflections
*a* = 11.2271 (19) Å	θ = 2.6–24.9°
*b* = 10.1360 (17) Å	µ = 0.35 mm^−^^1^
*c* = 25.660 (4) Å	*T* = 293 K
β = 102.233 (2)°	Prism, colorless
*V* = 2853.7 (8) Å^3^	0.32 × 0.12 × 0.10 mm
*Z* = 4	

### Data collection

**Table:**

Bruker SMART CCD area-detector diffractometer	5028 independent reflections
Radiation source: fine-focus sealed tube	3977 reflections with *I* > 2σ(*I*)
graphite	*R*_int_ = 0.021
φ and ω scans	θ_max_ = 25.0°, θ_min_ = 1.9°
Absorption correction: multi-scan (*SADABS*; Sheldrick, 1996)	*h* = −13→13
*T*_min_ = 0.771, *T*_max_ = 1.000	*k* = −12→9
15046 measured reflections	*l* = −30→30

### Refinement

**Table:**

Refinement on *F*^2^	Primary atom site location: structure-invariant direct methods
Least-squares matrix: full	Secondary atom site location: difference Fourier map
*R*[*F*^2^ > 2σ(*F*^2^)] = 0.046	Hydrogen site location: inferred from neighbouring sites
*wR*(*F*^2^) = 0.137	H-atom parameters constrained
*S* = 1.06	*w* = 1/[σ^2^(*F*_o_^2^) + (0.0698*P*)^2^ + 1.7665*P*] where *P* = (*F*_o_^2^ + 2*F*_c_^2^)/3
5028 reflections	(Δ/σ)_max_ = 0.001
373 parameters	Δρ_max_ = 0.78 e Å^−^^3^
0 restraints	Δρ_min_ = −0.45 e Å^−^^3^

### Special details

**Table:**

Geometry. All e.s.d.'s (except the e.s.d. in the dihedral angle between two l.s. planes) are estimated using the full covariance matrix. The cell e.s.d.'s are taken into account individually in the estimation of e.s.d.'s in distances, angles and torsion angles; correlations between e.s.d.'s in cell parameters are only used when they are defined by crystal symmetry. An approximate (isotropic) treatment of cell e.s.d.'s is used for estimating e.s.d.'s involving l.s. planes.
Refinement. Refinement of *F*^2^ against ALL reflections. The weighted *R*-factor *wR* and goodness of fit *S* are based on *F*^2^, conventional *R*-factors *R* are based on *F*, with *F* set to zero for negative *F*^2^. The threshold expression of *F*^2^ > σ(*F*^2^) is used only for calculating *R*-factors(gt) *etc*. and is not relevant to the choice of reflections for refinement. *R*-factors based on *F*^2^ are statistically about twice as large as those based on *F*, and *R*- factors based on ALL data will be even larger.

### Fractional atomic coordinates and isotropic or equivalent isotropic displacement parameters (Å^2^)

**Table:**

	*x*	*y*	*z*	*U*_iso_*/*U*_eq_	
Cl1	−0.00213 (9)	0.49776 (9)	0.40638 (4)	0.0728 (3)	
Cl2	−0.11702 (9)	0.58796 (9)	0.15026 (4)	0.0810 (3)	
S1	0.14276 (6)	0.07999 (6)	0.09665 (2)	0.03344 (17)	
O1	0.06429 (15)	0.32169 (19)	0.17809 (6)	0.0420 (4)	
O2	0.11152 (17)	0.45234 (18)	0.07819 (8)	0.0491 (5)	
O3	0.0674 (2)	−0.1707 (2)	0.14149 (9)	0.0608 (6)	
O4	−0.1101 (2)	−0.0950 (2)	0.14492 (11)	0.0752 (7)	
N1	−0.03379 (17)	0.2563 (2)	0.09591 (7)	0.0325 (4)	
N2	0.08279 (17)	0.23245 (19)	0.08582 (7)	0.0310 (4)	
N3	0.21131 (18)	0.0735 (2)	0.16139 (7)	0.0333 (4)	
N4	0.2211 (2)	0.0837 (2)	0.24814 (8)	0.0418 (5)	
N5	0.0473 (2)	−0.0140 (2)	0.20121 (8)	0.0439 (5)	
N6	0.0006 (2)	−0.0955 (2)	0.15925 (9)	0.0476 (6)	
N7	0.1465 (2)	0.3052 (3)	0.40109 (9)	0.0525 (6)	
C1	−0.1896 (3)	0.4535 (3)	0.17016 (13)	0.0583 (8)	
C2	−0.2914 (3)	0.4740 (5)	0.19289 (16)	0.0796 (11)	
H2	−0.3207	0.5589	0.1958	0.095*	
C3	−0.3459 (4)	0.3701 (5)	0.21039 (17)	0.0872 (13)	
H3	−0.4121	0.3841	0.2262	0.105*	
C4	−0.3057 (3)	0.2426 (5)	0.20542 (15)	0.0795 (11)	
H4	−0.3468	0.1727	0.2171	0.095*	
C5	−0.2077 (2)	0.2171 (4)	0.18388 (12)	0.0620 (9)	
H5	−0.1792	0.1314	0.1820	0.074*	
C6	−0.1483 (2)	0.3285 (3)	0.16371 (10)	0.0429 (6)	
C7	−0.0311 (2)	0.3051 (2)	0.14593 (9)	0.0333 (5)	
C8	−0.1401 (2)	0.2467 (3)	0.04779 (9)	0.0387 (6)	
C9	−0.2336 (3)	0.1494 (4)	0.05798 (15)	0.0883 (14)	
H9A	−0.2744	0.1846	0.0842	0.132*	
H9B	−0.1941	0.0681	0.0708	0.132*	
H9C	−0.2920	0.1334	0.0254	0.132*	
C10	−0.1969 (3)	0.3813 (3)	0.03440 (14)	0.0670 (9)	
H10A	−0.2587	0.3756	0.0023	0.100*	
H10B	−0.1352	0.4427	0.0293	0.100*	
H10C	−0.2327	0.4110	0.0631	0.100*	
C11	−0.0898 (3)	0.2030 (5)	−0.00012 (13)	0.0930 (15)	
H11A	−0.0538	0.1171	0.0066	0.139*	
H11B	−0.0292	0.2647	−0.0060	0.139*	
H11C	−0.1549	0.1996	−0.0311	0.139*	
C12	0.1470 (2)	0.3426 (2)	0.07288 (9)	0.0345 (5)	
C13	0.2546 (2)	0.3156 (3)	0.04891 (10)	0.0387 (6)	
C14	0.2425 (3)	0.2453 (3)	0.00197 (12)	0.0538 (7)	
H14	0.1692	0.2035	−0.0124	0.065*	
C15	0.3376 (3)	0.2368 (4)	−0.02354 (13)	0.0677 (9)	
H15	0.3278	0.1915	−0.0557	0.081*	
C16	0.4471 (3)	0.2949 (4)	−0.00196 (15)	0.0699 (10)	
H16	0.5123	0.2870	−0.0188	0.084*	
C17	0.4599 (3)	0.3646 (4)	0.04451 (15)	0.0670 (9)	
H17	0.5342	0.4041	0.0592	0.080*	
C18	0.3636 (2)	0.3768 (3)	0.06963 (12)	0.0504 (7)	
H18	0.3724	0.4265	0.1007	0.061*	
C19	0.3185 (2)	0.1561 (3)	0.18253 (10)	0.0453 (6)	
H19A	0.3066	0.2453	0.1687	0.054*	
H19B	0.3912	0.1195	0.1733	0.054*	
C20	0.3281 (3)	0.1541 (4)	0.24168 (12)	0.0633 (9)	
H20A	0.4015	0.1088	0.2596	0.076*	
H20B	0.3286	0.2429	0.2558	0.076*	
C21	0.1539 (2)	0.0411 (2)	0.20220 (9)	0.0346 (5)	
C22	0.1974 (3)	0.0529 (3)	0.30041 (10)	0.0506 (7)	
H22A	0.2731	0.0267	0.3240	0.061*	
H22B	0.1417	−0.0212	0.2971	0.061*	
C23	0.1436 (2)	0.1681 (3)	0.32484 (9)	0.0400 (6)	
C24	0.1910 (3)	0.2073 (3)	0.37612 (10)	0.0467 (7)	
H24	0.2590	0.1626	0.3949	0.056*	
C25	0.0517 (3)	0.3670 (3)	0.37382 (11)	0.0464 (7)	
C26	−0.0045 (3)	0.3379 (4)	0.32266 (13)	0.0657 (9)	
H26	−0.0724	0.3849	0.3051	0.079*	
C27	0.0435 (3)	0.2360 (3)	0.29801 (12)	0.0628 (9)	
H27	0.0079	0.2132	0.2631	0.075*	

### Atomic displacement parameters (Å^2^)

**Table:**

	*U*^11^	*U*^22^	*U*^33^	*U*^12^	*U*^13^	*U*^23^
Cl1	0.0906 (6)	0.0576 (5)	0.0834 (6)	0.0160 (4)	0.0483 (5)	−0.0003 (4)
Cl2	0.0914 (7)	0.0443 (5)	0.1006 (7)	−0.0010 (4)	0.0053 (5)	−0.0035 (4)
S1	0.0415 (3)	0.0298 (3)	0.0286 (3)	0.0009 (3)	0.0063 (2)	−0.0033 (2)
O1	0.0343 (9)	0.0553 (12)	0.0340 (9)	0.0017 (8)	0.0014 (7)	−0.0058 (8)
O2	0.0548 (11)	0.0321 (11)	0.0659 (13)	−0.0003 (9)	0.0254 (10)	−0.0003 (9)
O3	0.0806 (15)	0.0374 (12)	0.0652 (13)	−0.0030 (11)	0.0173 (11)	−0.0078 (10)
O4	0.0554 (14)	0.0655 (16)	0.0953 (18)	−0.0118 (11)	−0.0055 (13)	−0.0019 (13)
N1	0.0277 (10)	0.0387 (11)	0.0301 (10)	−0.0007 (9)	0.0041 (8)	−0.0005 (8)
N2	0.0311 (10)	0.0308 (11)	0.0314 (10)	−0.0001 (8)	0.0074 (8)	0.0010 (8)
N3	0.0355 (10)	0.0325 (11)	0.0309 (10)	0.0015 (9)	0.0043 (8)	−0.0018 (8)
N4	0.0521 (13)	0.0433 (13)	0.0282 (10)	0.0020 (10)	0.0045 (9)	−0.0005 (9)
N5	0.0522 (14)	0.0419 (13)	0.0392 (12)	−0.0072 (11)	0.0133 (10)	0.0006 (10)
N6	0.0571 (15)	0.0339 (13)	0.0494 (13)	−0.0090 (11)	0.0056 (12)	0.0077 (10)
N7	0.0586 (15)	0.0557 (15)	0.0410 (12)	0.0078 (12)	0.0057 (11)	−0.0084 (11)
C1	0.0517 (17)	0.059 (2)	0.0607 (19)	0.0063 (15)	0.0032 (14)	−0.0145 (15)
C2	0.063 (2)	0.097 (3)	0.079 (2)	0.018 (2)	0.0165 (19)	−0.030 (2)
C3	0.062 (2)	0.121 (4)	0.085 (3)	0.017 (2)	0.029 (2)	−0.018 (3)
C4	0.063 (2)	0.109 (3)	0.070 (2)	−0.012 (2)	0.0231 (18)	0.011 (2)
C5	0.0303 (14)	0.093 (3)	0.0626 (19)	−0.0023 (15)	0.0103 (13)	−0.0202 (18)
C6	0.0368 (14)	0.0541 (17)	0.0365 (13)	0.0026 (12)	0.0052 (11)	−0.0059 (12)
C7	0.0357 (13)	0.0306 (13)	0.0335 (12)	−0.0004 (10)	0.0071 (10)	0.0015 (10)
C8	0.0350 (13)	0.0464 (15)	0.0307 (12)	−0.0004 (11)	−0.0019 (10)	0.0003 (11)
C9	0.080 (2)	0.093 (3)	0.073 (2)	−0.048 (2)	−0.0287 (19)	0.026 (2)
C10	0.0602 (19)	0.063 (2)	0.066 (2)	0.0062 (16)	−0.0140 (16)	0.0131 (16)
C11	0.064 (2)	0.161 (4)	0.0438 (18)	0.030 (2)	−0.0115 (16)	−0.039 (2)
C12	0.0366 (13)	0.0347 (14)	0.0323 (12)	−0.0034 (11)	0.0071 (10)	−0.0013 (10)
C13	0.0420 (14)	0.0359 (14)	0.0407 (13)	−0.0006 (11)	0.0141 (11)	0.0046 (11)
C14	0.0537 (18)	0.060 (2)	0.0520 (17)	−0.0064 (14)	0.0199 (14)	−0.0092 (14)
C15	0.071 (2)	0.084 (3)	0.0565 (19)	0.0039 (19)	0.0341 (17)	−0.0095 (17)
C16	0.060 (2)	0.081 (3)	0.081 (2)	0.0067 (18)	0.0424 (18)	0.005 (2)
C17	0.0424 (17)	0.079 (2)	0.084 (2)	−0.0078 (16)	0.0235 (16)	−0.003 (2)
C18	0.0431 (15)	0.0540 (18)	0.0561 (17)	−0.0040 (13)	0.0150 (13)	−0.0050 (14)
C19	0.0390 (14)	0.0513 (17)	0.0442 (15)	−0.0039 (12)	0.0054 (11)	−0.0089 (12)
C20	0.062 (2)	0.078 (2)	0.0435 (16)	−0.0180 (17)	−0.0028 (14)	−0.0057 (15)
C21	0.0444 (14)	0.0271 (12)	0.0311 (12)	0.0074 (10)	0.0054 (10)	0.0025 (9)
C22	0.076 (2)	0.0428 (16)	0.0308 (13)	0.0079 (14)	0.0067 (13)	0.0051 (11)
C23	0.0505 (15)	0.0385 (15)	0.0303 (12)	0.0006 (12)	0.0072 (11)	0.0032 (10)
C24	0.0507 (16)	0.0485 (17)	0.0375 (14)	0.0095 (13)	0.0020 (12)	−0.0009 (12)
C25	0.0529 (16)	0.0415 (15)	0.0501 (16)	0.0011 (13)	0.0228 (13)	0.0040 (12)
C26	0.063 (2)	0.073 (2)	0.0566 (19)	0.0275 (17)	0.0018 (15)	0.0078 (17)
C27	0.072 (2)	0.073 (2)	0.0358 (15)	0.0169 (17)	−0.0070 (14)	−0.0010 (14)

### Geometric parameters (Å, °)

**Table:**

Cl1—C25	1.742 (3)	C9—H9B	0.9600
Cl2—C1	1.721 (4)	C9—H9C	0.9600
S1—N3	1.6777 (19)	C10—H10A	0.9600
S1—N2	1.685 (2)	C10—H10B	0.9600
O1—C7	1.218 (3)	C10—H10C	0.9600
O2—C12	1.199 (3)	C11—H11A	0.9600
O3—N6	1.224 (3)	C11—H11B	0.9600
O4—N6	1.218 (3)	C11—H11C	0.9600
N1—C7	1.370 (3)	C12—C13	1.493 (3)
N1—N2	1.408 (3)	C13—C18	1.375 (4)
N1—C8	1.527 (3)	C13—C14	1.381 (4)
N2—C12	1.407 (3)	C14—C15	1.368 (4)
N3—C21	1.381 (3)	C14—H14	0.9300
N3—C19	1.471 (3)	C15—C16	1.370 (5)
N4—C21	1.330 (3)	C15—H15	0.9300
N4—C20	1.435 (4)	C16—C17	1.367 (5)
N4—C22	1.456 (3)	C16—H16	0.9300
N5—C21	1.315 (3)	C17—C18	1.377 (4)
N5—N6	1.370 (3)	C17—H17	0.9300
N7—C25	1.304 (4)	C18—H18	0.9300
N7—C24	1.334 (4)	C19—C20	1.499 (4)
C1—C6	1.371 (4)	C19—H19A	0.9700
C1—C2	1.403 (5)	C19—H19B	0.9700
C2—C3	1.342 (6)	C20—H20A	0.9700
C2—H2	0.9300	C20—H20B	0.9700
C3—C4	1.384 (6)	C22—C23	1.510 (4)
C3—H3	0.9300	C22—H22A	0.9700
C4—C5	1.356 (5)	C22—H22B	0.9700
C4—H4	0.9300	C23—C24	1.370 (4)
C5—C6	1.461 (5)	C23—C27	1.372 (4)
C5—H5	0.9300	C24—H24	0.9300
C6—C7	1.500 (3)	C25—C26	1.363 (4)
C8—C9	1.503 (4)	C26—C27	1.379 (5)
C8—C10	1.515 (4)	C26—H26	0.9300
C8—C11	1.523 (4)	C27—H27	0.9300
C9—H9A	0.9600		
			
N3—S1—N2	106.41 (10)	C8—C11—H11C	109.5
C7—N1—N2	113.44 (18)	H11A—C11—H11C	109.5
C7—N1—C8	129.7 (2)	H11B—C11—H11C	109.5
N2—N1—C8	115.95 (18)	O2—C12—N2	120.7 (2)
C12—N2—N1	116.74 (19)	O2—C12—C13	122.3 (2)
C12—N2—S1	124.08 (16)	N2—C12—C13	116.8 (2)
N1—N2—S1	118.79 (15)	C18—C13—C14	119.0 (3)
C21—N3—C19	109.44 (19)	C18—C13—C12	119.3 (2)
C21—N3—S1	124.76 (16)	C14—C13—C12	121.0 (2)
C19—N3—S1	120.71 (17)	C15—C14—C13	120.5 (3)
C21—N4—C20	113.0 (2)	C15—C14—H14	119.7
C21—N4—C22	124.5 (2)	C13—C14—H14	119.7
C20—N4—C22	122.2 (2)	C14—C15—C16	120.3 (3)
C21—N5—N6	117.8 (2)	C14—C15—H15	119.9
O4—N6—O3	123.8 (3)	C16—C15—H15	119.9
O4—N6—N5	115.5 (3)	C17—C16—C15	119.7 (3)
O3—N6—N5	120.4 (2)	C17—C16—H16	120.2
C25—N7—C24	116.5 (2)	C15—C16—H16	120.2
C6—C1—C2	120.9 (3)	C16—C17—C18	120.4 (3)
C6—C1—Cl2	120.2 (2)	C16—C17—H17	119.8
C2—C1—Cl2	119.0 (3)	C18—C17—H17	119.8
C3—C2—C1	119.5 (4)	C13—C18—C17	120.1 (3)
C3—C2—H2	120.3	C13—C18—H18	119.9
C1—C2—H2	120.3	C17—C18—H18	119.9
C2—C3—C4	121.4 (4)	N3—C19—C20	104.1 (2)
C2—C3—H3	119.3	N3—C19—H19A	110.9
C4—C3—H3	119.3	C20—C19—H19A	110.9
C5—C4—C3	121.6 (4)	N3—C19—H19B	110.9
C5—C4—H4	119.2	C20—C19—H19B	110.9
C3—C4—H4	119.2	H19A—C19—H19B	109.0
C4—C5—C6	117.9 (3)	N4—C20—C19	104.0 (2)
C4—C5—H5	121.1	N4—C20—H20A	111.0
C6—C5—H5	121.1	C19—C20—H20A	111.0
C1—C6—C5	118.7 (3)	N4—C20—H20B	111.0
C1—C6—C7	121.6 (3)	C19—C20—H20B	111.0
C5—C6—C7	118.8 (2)	H20A—C20—H20B	109.0
O1—C7—N1	121.8 (2)	N5—C21—N4	119.9 (2)
O1—C7—C6	118.3 (2)	N5—C21—N3	131.0 (2)
N1—C7—C6	119.7 (2)	N4—C21—N3	109.0 (2)
C9—C8—C10	110.7 (3)	N4—C22—C23	112.6 (2)
C9—C8—C11	110.0 (3)	N4—C22—H22A	109.1
C10—C8—C11	106.8 (3)	C23—C22—H22A	109.1
C9—C8—N1	110.9 (2)	N4—C22—H22B	109.1
C10—C8—N1	110.2 (2)	C23—C22—H22B	109.1
C11—C8—N1	108.2 (2)	H22A—C22—H22B	107.8
C8—C9—H9A	109.5	C24—C23—C27	116.5 (3)
C8—C9—H9B	109.5	C24—C23—C22	121.0 (2)
H9A—C9—H9B	109.5	C27—C23—C22	122.5 (2)
C8—C9—H9C	109.5	N7—C24—C23	124.7 (3)
H9A—C9—H9C	109.5	N7—C24—H24	117.7
H9B—C9—H9C	109.5	C23—C24—H24	117.7
C8—C10—H10A	109.5	N7—C25—C26	124.9 (3)
C8—C10—H10B	109.5	N7—C25—Cl1	115.8 (2)
H10A—C10—H10B	109.5	C26—C25—Cl1	119.3 (2)
C8—C10—H10C	109.5	C25—C26—C27	117.2 (3)
H10A—C10—H10C	109.5	C25—C26—H26	121.4
H10B—C10—H10C	109.5	C27—C26—H26	121.4
C8—C11—H11A	109.5	C23—C27—C26	120.3 (3)
C8—C11—H11B	109.5	C23—C27—H27	119.9
H11A—C11—H11B	109.5	C26—C27—H27	119.9
			
C7—N1—N2—C12	−79.1 (2)	N2—C12—C13—C18	−130.5 (3)
C8—N1—N2—C12	91.0 (2)	O2—C12—C13—C14	−116.9 (3)
C7—N1—N2—S1	94.0 (2)	N2—C12—C13—C14	58.8 (3)
C8—N1—N2—S1	−95.8 (2)	C18—C13—C14—C15	0.0 (5)
N3—S1—N2—C12	86.82 (19)	C12—C13—C14—C15	170.8 (3)
N3—S1—N2—N1	−85.75 (17)	C13—C14—C15—C16	1.8 (5)
N2—S1—N3—C21	86.2 (2)	C14—C15—C16—C17	−1.8 (6)
N2—S1—N3—C19	−65.8 (2)	C15—C16—C17—C18	−0.1 (6)
C21—N5—N6—O4	−146.8 (2)	C14—C13—C18—C17	−1.8 (4)
C21—N5—N6—O3	39.2 (3)	C12—C13—C18—C17	−172.8 (3)
C6—C1—C2—C3	2.6 (5)	C16—C17—C18—C13	1.9 (5)
Cl2—C1—C2—C3	−177.5 (3)	C21—N3—C19—C20	7.4 (3)
C1—C2—C3—C4	−1.5 (6)	S1—N3—C19—C20	163.2 (2)
C2—C3—C4—C5	1.7 (6)	C21—N4—C20—C19	2.2 (3)
C3—C4—C5—C6	−2.7 (5)	C22—N4—C20—C19	176.7 (2)
C2—C1—C6—C5	−3.6 (4)	N3—C19—C20—N4	−5.6 (3)
Cl2—C1—C6—C5	176.5 (2)	N6—N5—C21—N4	−157.9 (2)
C2—C1—C6—C7	−172.2 (3)	N6—N5—C21—N3	27.1 (4)
Cl2—C1—C6—C7	7.8 (4)	C20—N4—C21—N5	−173.5 (3)
C4—C5—C6—C1	3.6 (4)	C22—N4—C21—N5	12.1 (4)
C4—C5—C6—C7	172.6 (3)	C20—N4—C21—N3	2.5 (3)
N2—N1—C7—O1	−1.8 (3)	C22—N4—C21—N3	−171.9 (2)
C8—N1—C7—O1	−170.3 (2)	C19—N3—C21—N5	169.1 (3)
N2—N1—C7—C6	−176.7 (2)	S1—N3—C21—N5	14.4 (4)
C8—N1—C7—C6	14.9 (4)	C19—N3—C21—N4	−6.3 (3)
C1—C6—C7—O1	75.6 (3)	S1—N3—C21—N4	−160.98 (17)
C5—C6—C7—O1	−93.0 (3)	C21—N4—C22—C23	−105.2 (3)
C1—C6—C7—N1	−109.4 (3)	C20—N4—C22—C23	80.9 (3)
C5—C6—C7—N1	82.0 (3)	N4—C22—C23—C24	−130.6 (3)
C7—N1—C8—C9	−67.4 (4)	N4—C22—C23—C27	51.4 (4)
N2—N1—C8—C9	124.4 (3)	C25—N7—C24—C23	−0.5 (5)
C7—N1—C8—C10	55.6 (3)	C27—C23—C24—N7	0.2 (5)
N2—N1—C8—C10	−112.7 (3)	C22—C23—C24—N7	−177.9 (3)
C7—N1—C8—C11	172.0 (3)	C24—N7—C25—C26	0.6 (5)
N2—N1—C8—C11	3.7 (3)	C24—N7—C25—Cl1	−178.1 (2)
N1—N2—C12—O2	10.9 (3)	N7—C25—C26—C27	−0.4 (5)
S1—N2—C12—O2	−161.8 (2)	Cl1—C25—C26—C27	178.2 (3)
N1—N2—C12—C13	−164.91 (19)	C24—C23—C27—C26	−0.1 (5)
S1—N2—C12—C13	22.4 (3)	C22—C23—C27—C26	178.0 (3)
O2—C12—C13—C18	53.8 (4)	C25—C26—C27—C23	0.1 (5)
